# Endocrine regimen and early subclinical atherosclerosis in premenopausal HR+/HER2− breast cancer: real-world evidence and regimen-dependent effects of Sanhuang Decoction

**DOI:** 10.3389/fonc.2026.1695776

**Published:** 2026-01-28

**Authors:** Ke Hong, Cong Wang, Menglei Yang, Mengyue Du, Huanhuan Wang, Jiawei Wu, Xinhe Liu, Xiaoyu Li, Wenjie Wu, Shuqin Li, Hongyu Cheng, Chang Yao

**Affiliations:** Department of Breast Surgery, Affiliated Hospital of Nanjing University of Chinese Medicine, Nanjing, China

**Keywords:** arterial plaque, breast cancer, endocrine therapy, lipid metabolism, Sanhuang Decoction, tamoxifen

## Abstract

**Introduction:**

Adjuvant endocrine therapy for hormone receptor–positive, human epidermal growth factor receptor 2–negative (HR+/HER2−) breast cancer improves survival but may worsen cardiometabolic health; the cardiovascular impact of aromatase inhibitor plus ovarian function suppression (AI+OFS) in premenopausal women remains unclear. We compared endocrine regimens on subclinical atherosclerosis and 24-month lipid trajectories and explored potential modulatory effects of Sanhuang Decoction (SHD).

**Methods:**

In this retrospective cohort of 280 HR+/HER2− patients, initial endocrine therapy was AI+OFS (n = 95), tamoxifen (TAM; n = 141), or TAM+OFS (n = 44). Lipid profiles (total cholesterol, triglycerides, low-density lipoprotein cholesterol [LDL-C], high-density lipoprotein cholesterol [HDL-C]) were analyzed using mixed-effects models adjusted for cardiometabolic and treatment covariates. New-onset or worsening fatty liver, new-onset carotid plaque, and initiation of lipid-lowering therapy were evaluated with Kaplan–Meier and Cox models.

**Results:**

In adjusted mixed-effects models, AI+OFS versus TAM was associated with higher LDL-C (β 0.088 mmol/L; 95% confidence interval [CI] 0.013–0.163) and lower HDL-C (β −0.046 mmol/L; 95% CI −0.090 to −0.003), whereas total cholesterol and triglycerides did not differ between regimens. Incidences of fatty liver, carotid plaque, and initiation of lipid-lowering therapy varied across groups, but most adjusted Cox models showed no significant regimen effects. AI+OFS showed a trend toward a higher hazard of new-onset carotid plaque versus TAM (hazard ratio [HR] 3.09; 95% CI 0.96–9.93), and higher baseline glycated hemoglobin predicted earlier initiation of lipid-lowering therapy (HR 2.54; 95% CI 1.29–4.99). In exploratory interaction analyses, among SHD users TAM+OFS versus TAM was associated with lower LDL-C and total cholesterol, whereas in AI+OFS versus TAM SHD use was associated with lower HDL-C.

**Conclusion:**

In premenopausal women with HR+/HER2− breast cancer, AI+OFS was associated with an adverse lipid profile and a possible increase in carotid plaque compared with TAM-based regimens. Regimen-dependent associations between SHD and lipid profiles support individualized cardiovascular monitoring and cautious use of adjunctive SHD in this population.

## 1 Introduction

Hormone receptor-positive (HR-positive), human epidermal growth factor receptor 2-negative (HER2−negative) breast cancer (BC) is the most common subtype, accounting for 65% to 75% of all cases. Adjuvant endocrine therapy (ET) is the standard treatment for early HR-positive BC, significantly reducing recurrence and improving overall survival (1). This therapy typically lasts 5 to 10 years, with long-term adherence being critical for optimal patient outcomes. Key ET agents include tamoxifen (TAM), a selective estrogen receptor modulator (SERM), and aromatase inhibitors (AIs) (2). For postmenopausal women, AIs are generally preferred due to superior clinical outcomes, while premenopausal women with high-risk disease often receive ovarian function suppression (OFS) combined with TAM or AI (3, 4).

Despite its oncological benefits, long-term ET can lead to significant metabolic and cardiovascular adverse effects, potentially impacting quality of life and treatment adherence (5). Estrogen plays a crucial role in lipid metabolism and cardiovascular health, and its suppression by ET can induce metabolic disturbances. Cardiovascular disease (CVD) is a leading cause of non-cancer-related mortality in BC survivors, particularly in older patients (6, 7).

Tamoxifen, while generally associated with favorable lipid changes such as reduced total cholesterol (TC) and low-density lipoprotein cholesterol (LDL-C) and increased apolipoprotein AI, can increase the risk of thromboembolic events like deep vein thrombosis and stroke (5, 8). Aromatase inhibitors, by contrast, are linked to hypercholesterolemia, myocardial infarction, and ischemic stroke in some studies (9, 10). However, recent large studies (11, 12) have presented conflicting evidence, suggesting AIs may not increase or even lower the risk of certain cardiovascular events. Ovarian function suppression, by causing a sharp decline in estrogen, can lead to an atherogenic lipid profile, including elevated TC, LDL-C, and triglycerides (TG), and unfavorable HDL-C changes (6, 13). It has also been associated with higher blood pressure and glucose risks when combined with tamoxifen. Non-alcoholic fatty liver disease (NAFLD) is another potential metabolic complication, linked to long-term estrogen suppression and metabolic syndrome (14, 15). However, most systematic reviews and large population-based studies of endocrine therapy–related cardiometabolic toxicity have predominantly included postmenopausal women and focused on overt cardiovascular events (e.g., myocardial infarction, heart failure, or cardiovascular death), rather than early subclinical vascular or hepatic changes (11, 12, 16, 17).

Because endocrine therapy is prolonged and data on its cardiovascular effects in premenopausal women remain limited, particularly for intensive regimens such as aromatase inhibitor plus ovarian function suppression (AI+OFS), the impact of these strategies on subclinical cardiovascular injury and atherosclerotic progression in younger breast cancer survivors is still poorly defined (16, 17). While landmark trials have established the oncologic efficacy of AI+OFS, the long-term cardiometabolic sequelae, particularly the risk of accelerated subclinical atherosclerosis in this demographic with decades of life expectancy post-treatment, remain poorly quantified in real-world settings (18). This study, therefore, aims to evaluate the differential impact of various adjuvant ET regimens (AI+OFS, TAM, and TAM+OFS) on longitudinal lipid profiles and, critically, on the risk of new-onset subclinical cardiometabolic events—including fatty liver, arterial plaque, and initiation of lipid-lowering therapy—in a real-world cohort of premenopausal HR-positive/HER2−negative patients. Additionally, in an exploratory analysis, we investigated the potential modulating effects of concomitant Sanhuang Decoction (SHD), a Traditional Chinese Medicine (TCM) formulation, on these cardiometabolic outcomes. Our prior research suggests that SHD exerts multi-target systemic regulatory effects, including modulation of inflammation and oxidative stress (19, 20) providing a rationale to explore whether it may also mitigate the specific cardiometabolic toxicities induced by different endocrine regimens, and to generate hypotheses for personalized integrative oncology.

## 2 Methods

### 2.1 Study design and participants

This retrospective single-center cohort included female patients with primary, non-metastatic, hormone receptor–positive (HR-positive), human epidermal growth factor receptor 2–negative (HER2-negative) breast cancer who initiated adjuvant endocrine therapy at Jiangsu Provincial Hospital of Chinese Medicine between January 2018 and December 2022. Eligible patients had electronic medical records with baseline laboratory data at endocrine therapy initiation (month 0) and at least one follow-up lipid assessment within 24 months. Exclusion criteria were stage IV disease at diagnosis, documented use of any lipid-lowering medication before the first endocrine therapy prescription, and missing baseline lipid data. According to the initial endocrine regimen, patients were categorized into three groups: tamoxifen alone (TAM), aromatase inhibitor plus ovarian function suppression (AI+OFS), and tamoxifen plus OFS (TAM+OFS). In routine practice during the study period, AI+OFS was preferentially prescribed to patients with higher clinicopathologic risk or relative contraindications to long-term tamoxifen (such as prior venous thromboembolism, endometrial pathology, or a desire for pregnancy), whereas TAM or TAM+OFS was generally used in lower-risk or tamoxifen-tolerant patients. After application of these criteria and exclusion of patients with pre-existing lipid-lowering therapy, 280 patients were included in the final analysis; no formal sample size calculation was performed, and all consecutive eligible patients during the study period were enrolled. The study was approved by the institutional ethics committee, and the requirement for written informed consent was waived because of the retrospective, non-interventional design.

### 2.2 Data collection and definitions

Clinical, pathological, treatment, and laboratory data were extracted from electronic records. The index date (baseline, month 0) was defined as the date of first prescription of the qualifying endocrine regimen. Baseline variables included age, body mass index (BMI), glycated hemoglobin (HbA1c), fasting plasma glucose, hepatic status (fatty liver), arterial plaque, cardiovascular risk factors, and tumor and treatment characteristics. Longitudinal lipid measurements (total cholesterol [TC], triglycerides [TG], low-density lipoprotein cholesterol [LDL-C], and high-density lipoprotein cholesterol [HDL-C]) were abstracted at baseline and at approximately 3-month intervals up to 24 months. Non-numeric laboratory results (e.g. values reported below the lower limit of detection) were imputed as half the lower limit.

Three time-to-event endpoints were defined: (1) new-onset or worsening fatty liver, based on ultrasound evidence of hepatic steatosis documented in radiology reports together with physician diagnosis and, when noted, in the absence of alternative secondary causes; (2) new-onset arterial plaque, defined as the first documented plaque on carotid or other arterial ultrasonography; and (3) initiation of lipid-lowering therapy, defined as the first prescription of a statin, fibrate, or other lipid-lowering drug after endocrine therapy initiation and within 24 months. For arterial plaque, vascular ultrasound reports were programmatically screened using predefined keywords, and a 10% random sample of candidate events was manually adjudicated by two reviewers. Patients with prevalent fatty liver or plaque at baseline were excluded from the corresponding incident-event analyses. Baseline dyslipidemia was defined as TG ≥ 1.7 mmol/L, TC ≥ 5.2 mmol/L, LDL-C ≥ 3.4 mmol/L, or HDL-C < 1.0 mmol/L.

### 2.3 Traditional Chinese medicine intervention

Concomitant use of Sanhuang Decoction (SHD), a hospital-compounded decoction of Astragalus membranaceus, Rheum officinale, and Curcuma longa, was identified from medication orders and progress notes. SHD exposure was summarized as ever versus never use and as time-varying use for exploratory interaction analyses.

### 2.4 Statistical analysis

Analyses were performed in Python (version 3.11.9) using pandas, statsmodels, and lifelines. A two-sided P-value < 0.05 was considered statistically significant. Continuous variables were inspected with histograms and Q–Q plots. Approximately normal variables were summarized as mean ± standard deviation and compared with one-way analysis of variance; skewed variables, including BMI, were summarized as median (interquartile range) and compared with the Kruskal–Wallis test. Categorical variables were expressed as counts and percentages and compared using the χ² test or Fisher’s exact test, as appropriate. To evaluate longitudinal lipid trajectories, we constructed patient-level long data sets with repeated measurements of TC, TG, LDL-C, and HDL-C at each available time point. Linear mixed-effects models with patient-level random intercepts were used to estimate the fixed effects of time, treatment group, and their interaction (time × group). All models were adjusted for baseline age, BMI, HbA1c, fasting glucose, baseline fatty liver, baseline arterial plaque, history of hypertension and diabetes, and receipt of adjuvant chemotherapy and radiotherapy. Models were fitted using statsmodels mixed-effects routines with maximum likelihood; if a mixed-effects model failed to converge, an ordinary least squares model with cluster-robust standard errors at the patient level was used as a fallback. Time-to-event analyses for new-onset fatty liver, new-onset arterial plaque, and initiation of lipid-lowering therapy were conducted using Kaplan–Meier estimates and log-rank tests to compare endocrine regimens. Multivariable Cox proportional hazards models were then fitted to estimate hazard ratios (HRs) and 95% confidence intervals, adjusting for treatment group, baseline age, BMI, and HbA1c. Patients with the event of interest at or before baseline were excluded from the corresponding Cox models, and follow-up time was censored at 24 months. The proportional hazards assumption was assessed using Schoenfeld residuals. Exploratory analyses evaluated effect modification by baseline lipid status and by concomitant SHD use. For these analyses, interaction terms between time, treatment group, and baseline lipid status or SHD use were added to the mixed-effects models. These exploratory analyses were considered hypothesis-generating, and no formal adjustment was made for multiple comparisons.

## 3 Results

### 3.1 Baseline characteristics

A total of 280 premenopausal patients with HR-positive/HER2-negative early breast cancer were included, comprising 95 in the AI+OFS group, 141 in the TAM group, and 44 in the TAM+OFS group (**Table 1**). The median age was 44 years (IQR, 39–49), and the median BMI was 23.44 kg/m² (IQR, 22.01–24.87). Age differed significantly across regimens (41 [37–46] vs. 48 [42–53] vs. 40 [38–45] years for AI+OFS, TAM, and TAM+OFS, respectively; *P* < 0.001), as did BMI (23.11 vs. 23.85 vs. 23.18 kg/m²; *P* = 0.0422) and HbA1c (5.60 vs. 5.69 vs. 5.53%; *P* < 0.01).

**Table 1:** Baseline demographic, cardiometabolic, tumor, and treatment characteristics by endocrine regimen.

Variable	Overall (n = 280)	AI+OFS (n = 95)	TAM (n = 141)	TAM+OFS (n = 44)	P value
Demographic and metabolic characteristics					
Age, years	44 (39–49)	41 (37–46)	48 (42–53)	40 (38–45)	<0.001
BMI, kg/m²	23.44 (22.01–24.87)	23.11 (21.42–24.40)	23.85 (22.54–24.96)	23.18 (20.70–26.05)	0.0422
HbA1c, %	5.62 (5.39–5.86)	5.60 (5.35–5.81)	5.69 (5.40–5.91)	5.53 (5.20–5.69)	<0.01
Fasting glucose, mmol/L	5.13 (4.65–5.41)	5.16 (4.96–5.41)	5.12 (4.61–5.45)	5.03 (4.51–5.27)	0.0605
AST, U/L	23.74 (19.00–25.51)	23.57 (21.74–25.07)	23.63 (18.00–25.33)	23.99 (21.25–29.21)	0.4577
ALT, U/L	23.15 (18.00–25.56)	23.00 (20.41–24.44)	23.20 (17.00–25.74)	23.23 (21.09–33.25)	0.5168
Cardiovascular risk factors and baseline organ status					
Smoking status					
Current	10 (3.6)	3 (3.2)	6 (4.3)	1 (2.3)	0.5579
Former	16 (5.7)	8 (8.4)	5 (3.5)	3 (6.8)	
Alcohol use	39 (13.9)	14 (14.7)	19 (13.5)	6 (13.6)	0.9612
Family history of CVD	46 (16.4)	16 (16.8)	22 (15.6)	8 (18.2)	0.9138
History of hypertension	25 (8.9)	2 (2.1)	15 (10.6)	8 (18.2)	<0.01
History of diabetes mellitus	13 (4.6)	2 (2.1)	8 (5.7)	3 (6.8)	0.3345
History of VTE	3 (1.1)	3 (3.2)	0 (0.0)	0 (0.0)	0.0522
Endometrial disease					
Hyperplasia	16 (5.7)	7 (7.4)	9 (6.4)	0 (0.0)	0.2619
Cancer	1 (0.4)	1 (1.1)	0 (0.0)	0 (0.0)	
Baseline fatty liver					
Mild	60 (21.4)	16 (16.8)	35 (24.8)	9 (20.5)	0.5646
Moderate–severe	23 (8.2)	7 (7.4)	11 (7.8)	5 (11.4)	
Baseline arterial plaque	61 (21.8)	18 (18.9)	37 (26.2)	6 (13.6)	0.1491
Tumor characteristics					
Histologic type					
Invasive ductal carcinoma	250 (89.3)	83 (87.4)	129 (91.5)	38 (86.4)	0.4787
Other	30 (10.7)	12 (12.6)	12 (8.5)	6 (13.6)	
Histologic grade					
Grade 1	44 (15.7)	6 (6.3)	31 (22.0)	7 (15.9)	<0.001
Grade 2	166 (59.3)	49 (51.6)	93 (66.0)	24 (54.5)	
Grade 3	70 (25.0)	40 (42.1)	17 (12.1)	13 (29.5)	
Ki-67 index, %					
≤20%	115 (41.1)	17 (17.9)	88 (62.4)	10 (22.7)	<0.001
>20%	165 (58.9)	78 (82.1)	53 (37.6)	34 (77.3)	
Pathologic T stage					
pT1	99 (35.4)	17 (17.9)	76 (53.9)	6 (13.6)	<0.001
pT2	126 (45.0)	44 (46.3)	55 (39.0)	27 (61.4)	
pT3	55 (19.6)	34 (35.8)	10 (7.1)	11 (25.0)	
Pathologic N stage					
pN0	178 (63.6)	40 (42.1)	117 (83.0)	21 (47.7)	<0.001
pN1	38 (13.6)	15 (15.8)	14 (9.9)	9 (20.5)	
pN2	51 (18.2)	32 (33.7)	7 (5.0)	12 (27.3)	
pN3	13 (4.6)	8 (8.4)	3 (2.1)	2 (4.5)	
Tumor size, cm	2.9 (1.6–4.6)	3.9 (2.3–6.2)	1.8 (1.3–3.2)	4.0 (2.8–5.0)	<0.001
Treatment characteristics					
Surgery type					
Mastectomy	170 (60.7)	74 (77.9)	65 (46.1)	31 (70.5)	<0.001
Breast-conserving	110 (39.3)	21 (22.1)	76 (53.9)	13 (29.5)	
Adjuvant chemotherapy	191 (68.2)	77 (81.1)	84 (59.6)	30 (68.2)	<0.01
Adjuvant radiotherapy	190 (67.9)	61 (64.2)	101 (71.6)	28 (63.6)	0.3947

Smoking status, alcohol use, and family history of cardiovascular disease were broadly comparable among groups (*P*>0.05), whereas a history of hypertension was less frequent in AI+OFS and more common in TAM and TAM+OFS (2.1% vs. 10.6% vs. 18.2%; *P* < 0.01). Baseline fatty liver and arterial plaque showed no statistically significant differences (*P* = 0.5646 and *P* = 0.1491).

Tumor characteristics were more advanced in the AI+OFS and TAM+OFS groups, with higher histologic grade, higher Ki-67 index, and more advanced pathologic T and N stages compared with TAM (*P* < 0.001). Consistently, mastectomy and adjuvant chemotherapy were more common in AI+OFS and TAM+OFS than in TAM (*P* < 0.001 and *P* < 0.01, respectively), whereas adjuvant radiotherapy use was similar across regimens (*P* = 0.3947).

### 3.2 Impact of endocrine therapies on longitudinal lipid profiles

Over 24 months of follow-up, median lipid levels remained relatively stable within each endocrine regimen (**Table 2**). At baseline, total cholesterol, triglycerides, LDL-C, and HDL-C were broadly comparable across the AI+OFS, TAM, and TAM+OFS groups (*P*>0.05). At 12 and 24 months, small between-group differences were observed for total cholesterol (*P* = 0.033 and P < 0.001, respectively) and triglycerides (both P < 0.001), but the median changes from baseline were close to zero in all regimens. For example, the 12-month change in triglycerides was −0.03 (−0.59–0.31), 0.03 (−0.16–0.72), and 0.05 (−0.26–0.47) mmol/L in the AI+OFS, TAM, and TAM+OFS groups, respectively (*P* = 0.019), and the corresponding 24-month changes were 0.00 (−0.68–0.38), 0.04 (−0.16–0.71), and 0.03 (−0.17–0.51) mmol/L (*P* = 0.133). Changes in LDL-C and HDL-C at 12 and 24 months did not differ significantly between regimens (*P*>0.05).

**Table 2:** Longitudinal lipid profiles over 24 months according to endocrine therapy regimen.

Variable	AI+OFS (n = 95)	TAM (n = 141)	TAM+OFS (n = 44)	P value
Total cholesterol, mmol/L				
Baseline	4.65 (4.53–4.77)	4.68 (4.50–4.76)	4.63 (4.32–4.81)	0.49
12 months	4.67 (4.60–4.75)	4.68 (4.63–4.74)	4.64 (4.46–4.68)	0.033
24 months	4.66 (4.60–4.71)	4.69 (4.65–4.74)	4.63 (4.61–4.67)	<0.001
Change (12 months − baseline)	0.04 (-0.12–0.35)	0.01 (-0.13–0.24)	-0.00 (-0.26–0.16)	0.57
Change (24 months − baseline)	0.01 (-0.23–0.18)	0.00 (-0.11–0.22)	0.00 (-0.20–0.23)	0.529
Triglycerides, mmol/L				
Baseline	1.70 (1.33–1.98)	1.68 (1.10–1.95)	1.72 (1.22–2.05)	0.81
12 months	1.70 (1.20–1.85)	1.82 (1.68–1.94)	1.76 (1.59–1.88)	<0.001
24 months	1.73 (1.46–1.82)	1.82 (1.70–1.93)	1.77 (1.68–1.86)	<0.001
Change (12 months − baseline)	-0.03 (-0.59–0.31)	0.03 (-0.16–0.72)	0.05 (-0.26–0.47)	0.019
Change (24 months − baseline)	0.00 (-0.68–0.38)	0.04 (-0.16–0.71)	0.03 (-0.17–0.51)	0.133
LDL-C, mmol/L				
Baseline	2.54 (2.44–2.70)	2.59 (2.43–2.69)	2.54 (2.36–2.64)	0.408
12 months	2.56 (2.51–2.73)	2.57 (2.52–2.61)	2.54 (2.43–2.57)	0.075
24 months	2.55 (2.50–2.62)	2.58 (2.52–2.61)	2.53 (2.49–2.56)	<0.01
Change (12 months − baseline)	0.01 (-0.16–0.30)	-0.01 (-0.19–0.15)	-0.00 (-0.25–0.06)	0.111
Change (24 months − baseline)	0.01 (-0.24–0.20)	-0.00 (-0.20–0.17)	-0.01 (-0.19–0.07)	0.495
HDL-C, mmol/L				
Baseline	1.50 (1.43–1.56)	1.49 (1.42–1.57)	1.50 (1.32–1.58)	0.653
12 months	1.50 (1.43–1.53)	1.50 (1.47–1.52)	1.51 (1.49–1.55)	0.07
24 months	1.50 (1.48–1.53)	1.50 (1.48–1.53)	1.52 (1.50–1.53)	0.051
Change (12 months − baseline)	-0.01 (-0.15–0.08)	-0.00 (-0.10–0.15)	0.02 (-0.06–0.25)	0.125
Change (24 months − baseline)	0.00 (-0.12–0.08)	0.00 (-0.06–0.16)	0.02 (-0.02–0.22)	0.091

**Figure 1:**
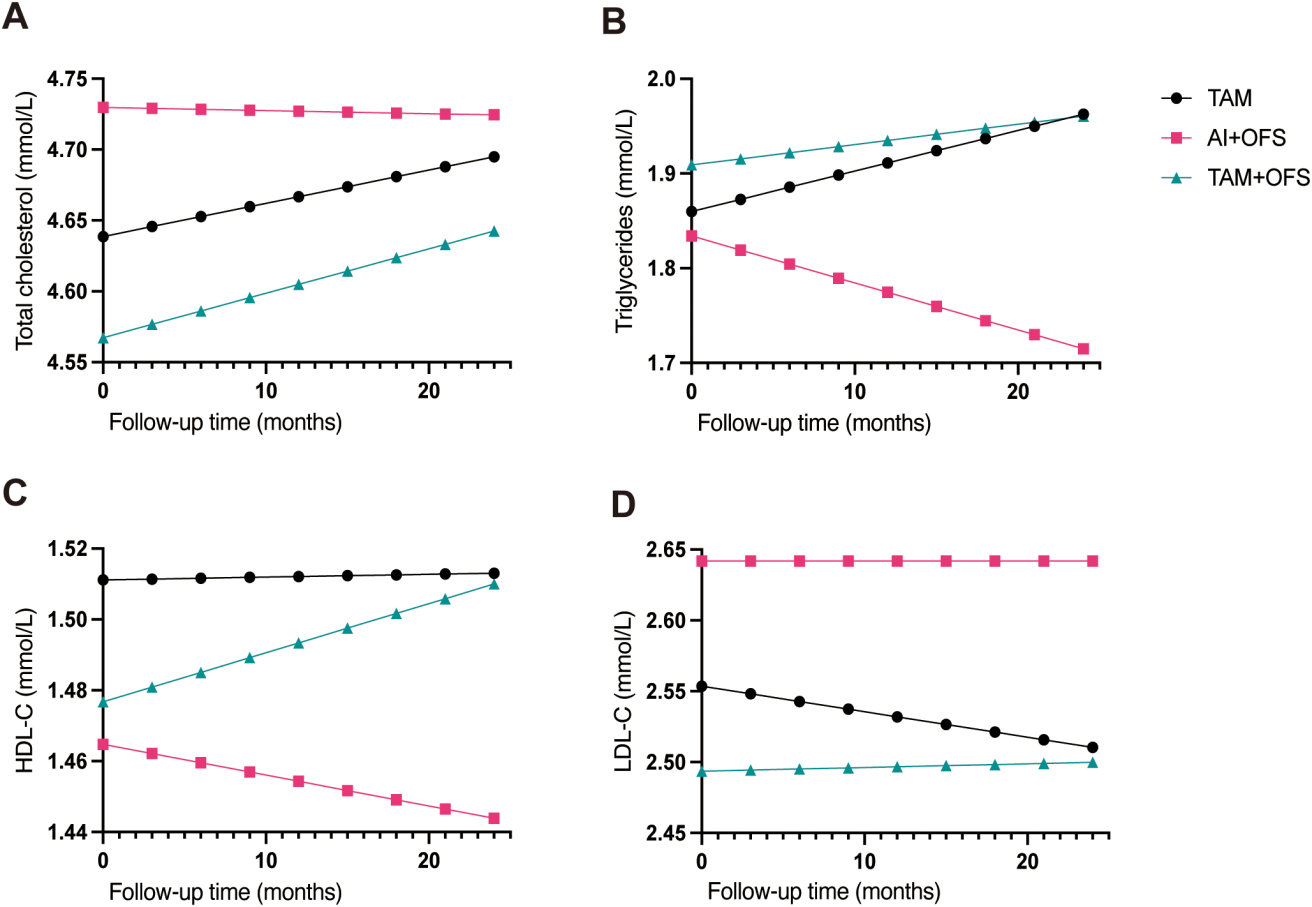
Model-adjusted lipid trajectories over 24 months by endocrine regimen. **(A)** Total cholesterol; **(B)** triglycerides; **(C)** HDL-C; **(D)** LDL-C. Curves show predicted mean lipid levels from linear mixed-effects models with fixed effects for time (months), regimen (AI+OFS, TAM, TAM+OFS), and their interaction, adjusted for baseline age, BMI, HbA1c, fasting glucose, fatty liver, arterial plaque, hypertension, diabetes, chemotherapy, and radiotherapy (TAM as reference; covariates held at medians/modes). If a mixed-effects model did not converge, linear regression with patient-level cluster-robust standard errors was used.

In linear mixed-effects models adjusting for baseline age, BMI, HbA1c, fasting glucose, baseline fatty liver, baseline arterial plaque, history of hypertension and diabetes, and adjuvant chemotherapy and radiotherapy, treatment regimen was associated with modest differences in LDL-C and HDL-C but not in triglycerides or total cholesterol (**Figure 1**). Compared with TAM, the AI+OFS group had higher LDL-C levels over time (β = 0.088 mmol/L; 95% CI, 0.013–0.163; *P* = 0.021), whereas TAM+OFS did not differ significantly (β = −0.060; 95% CI, −0.154–0.034; *P* = 0.211). For HDL-C, AI+OFS showed lower levels than TAM (β = −0.046 mmol/L; 95% CI, −0.090 to −0.003; *P* = 0.038), while TAM+OFS again was similar to TAM (β = −0.034; 95% CI, −0.090–0.021; *P* = 0.224). No statistically significant time-by-treatment interactions were detected for any lipid outcome (*P*>0.05).

### 3.3 Impact on risk of clinical events

Over 24 months, the crude incidence of metabolic and vascular events differed modestly across regimens (**Table 3**). New-onset or worsening fatty liver occurred in 23 of 95 patients (24.2%) in the AI+OFS group, 51 of 141 (36.2%) in the TAM group, and 20 of 44 (45.5%) in the TAM+OFS group (P = 0.031). New-onset carotid plaque was observed in 9 (9.5%), 3 (2.1%), and 3 (6.8%) patients, respectively (*P* = 0.044). Initiation of lipid-lowering therapy occurred in 14 (14.7%), 14 (9.9%), and 6 (13.6%) patients, with no significant between-group difference (*P* = 0.512).

**Table 3:** Incidence of metabolic and vascular events within 24 months according to endocrine therapy regimen.

Variable	AI+OFS (n = 95)	TAM (n = 141)	TAM+OFS (n = 44)	P value
New-onset or worsening fatty liver	23 (24.2%)	51 (36.2%)	20 (45.5%)	0.031
New-onset carotid plaque	9 (9.5%)	3 (2.1%)	3 (6.8%)	0.044
Initiation of lipid-lowering therapy	14 (14.7%)	14 (9.9%)	6 (13.6%)	0.512

**Figure 2:**
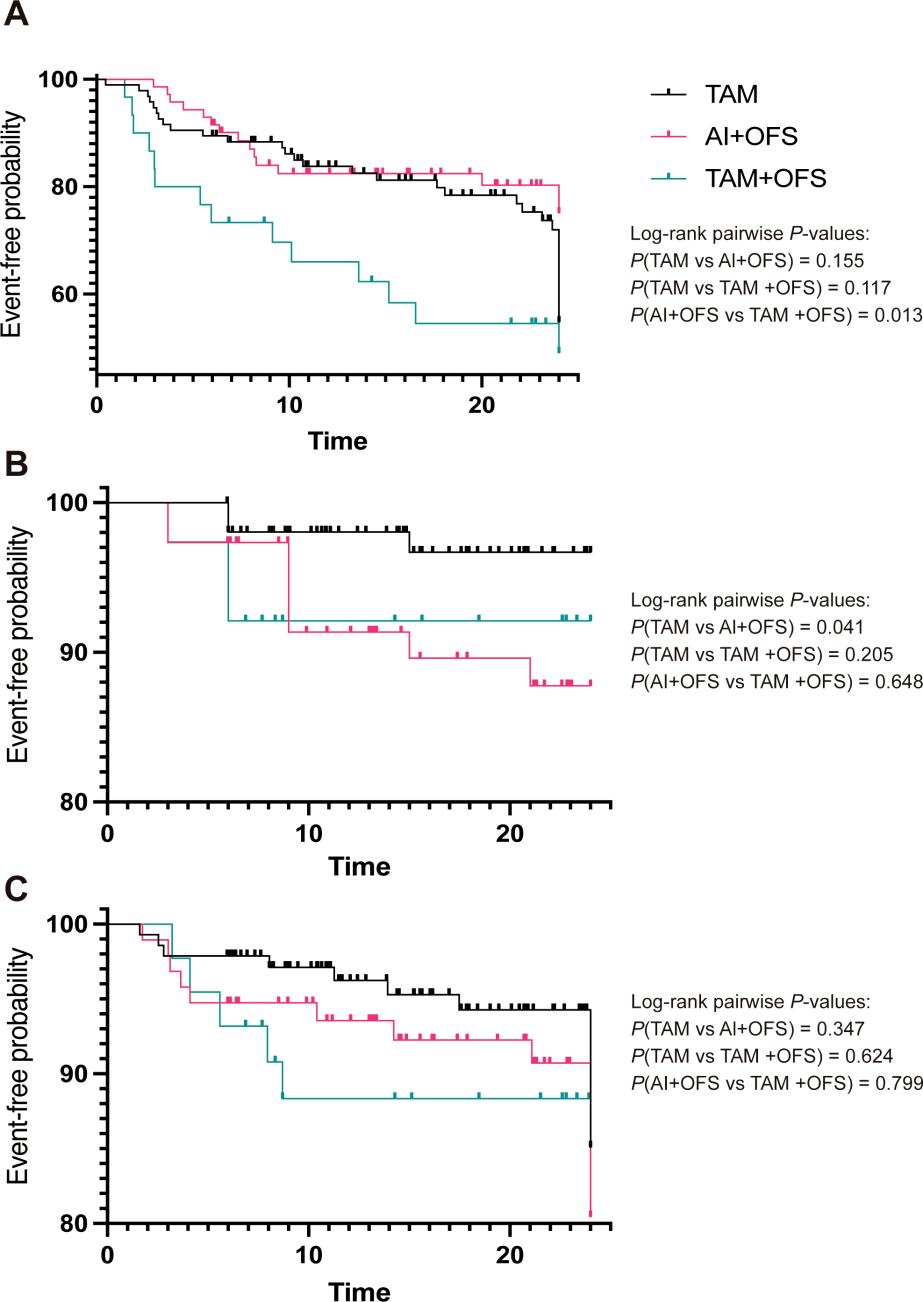
Kaplan–Meier curves for clinical events over 24 months by endocrine regimen. **(A)** New-onset or worsening fatty liver; **(B)** new-onset carotid plaque; **(C)** initiation of lipid-lowering therapy. Pairwise log-rank P values are shown in each panel. Hazard ratios were estimated using Cox models adjusted for baseline age, BMI, and HbA1c (Methods).

Kaplan–Meier analyses (**Figure 2**) showed no statistically significant pairwise differences in fatty liver-free survival between TAM and AI+OFS or TAM+OFS (log-rank P = 0.155 and 0.117, respectively), whereas AI+OFS had a lower fatty liver-free survival than TAM+OFS (log-rank *P* = 0.013). For new-onset carotid plaque, event-free survival was lower with AI+OFS than with TAM (log-rank *P* = 0.041), while comparisons involving TAM+OFS were not significant (all log-rank *P* ≥ 0.205). Kaplan–Meier curves for initiation of lipid-lowering therapy overlapped substantially, with all pairwise log-rank *P* ≥ 0.347.

In multivariable Cox models adjusting for baseline age, BMI, and HbA1c, no endocrine regimen or baseline covariate was significantly associated with new-onset or worsening fatty liver (*P*>0.05). For new-onset carotid plaque, the AI+OFS regimen showed a higher, though not statistically significant, hazard compared with TAM (HR 3.087; 95% CI, 0.960–9.930; P = 0.059), and baseline HbA1c was similarly close to significance (HR 2.579; 95% CI, 0.987–6.741; *P* = 0.053). For initiation of lipid-lowering therapy, hazards did not differ significantly by regimen (HR 1.920; 95% CI, 0.882–4.180; P = 0.100 for AI+OFS vs. TAM; HR 1.662; 95% CI, 0.619–4.462; *P* = 0.314 for TAM+OFS vs. TAM), whereas higher baseline HbA1c was independently associated with earlier treatment initiation (HR 2.537; 95% CI, 1.290–4.987; *P* = 0.007). These event patterns occurred in the context of modestly higher LDL-C and lower HDL-C levels over time in the AI+OFS group compared with TAM in the LMM analyses (**Supplementary Table S2**).

### 3.4 Exploratory analyses of effect modification

To explore potential factors that might modify the effects of endocrine therapy on lipid trajectories, a series of pre-specified exploratory interaction analyses were conducted. We specifically investigated the influence of two key variables: the patients’ baseline lipid status and the concomitant use of SHD.

#### 3.4.1 Effect modification by baseline lipid status

At baseline, nearly half of the cohort had hypertriglyceridaemia (triglycerides ≥1.7 mmol/L; 139/280, 49.6%), whereas high LDL-C (≥3.4 mmol/L), low HDL-C (<1.0 mmol/L), and high total cholesterol (≥5.2 mmol/L) were less frequent (12/280 [4.3%], 5/280 [1.8%], and 18/280 [6.4%], respectively). Any dyslipidaemia (≥1 abnormal lipid parameter) was present in 153 of 280 patients (54.6%). The prevalence of each abnormality was similar across the AI+OFS, TAM, and TAM+OFS groups (e.g. high triglycerides in 51.6%, 47.5%, and 52.3%, respectively; all *P* ≥ 0.3658; **Table 4**).

**Table 4:** Baseline lipid abnormalities according to endocrine therapy regimen.

Variable	Overall n (%)	TAM n (%)	AI+OFS n (%)	TAM+OFS n (%)	P value
High triglycerides (≥1.7 mmol/L)	139 (49.6)	67 (47.5)	49 (51.6)	23 (52.3)	0.7715
High LDL-C (≥3.4 mmol/L)	12 (4.3)	7 (5.0)	3 (3.2)	2 (4.5)	0.7945
Low HDL-C (<1.0 mmol/L)	5 (1.8)	1 (0.7)	3 (3.2)	1 (2.3)	0.3658
High total cholesterol (≥5.2 mmol/L)	18 (6.4)	9 (6.4)	5 (5.3)	4 (9.1)	0.6930
Any dyslipidaemia (≥1 abnormal parameter)	153 (54.6)	74 (52.5)	54 (56.8)	25 (56.8)	0.7653

**Figure 3:**
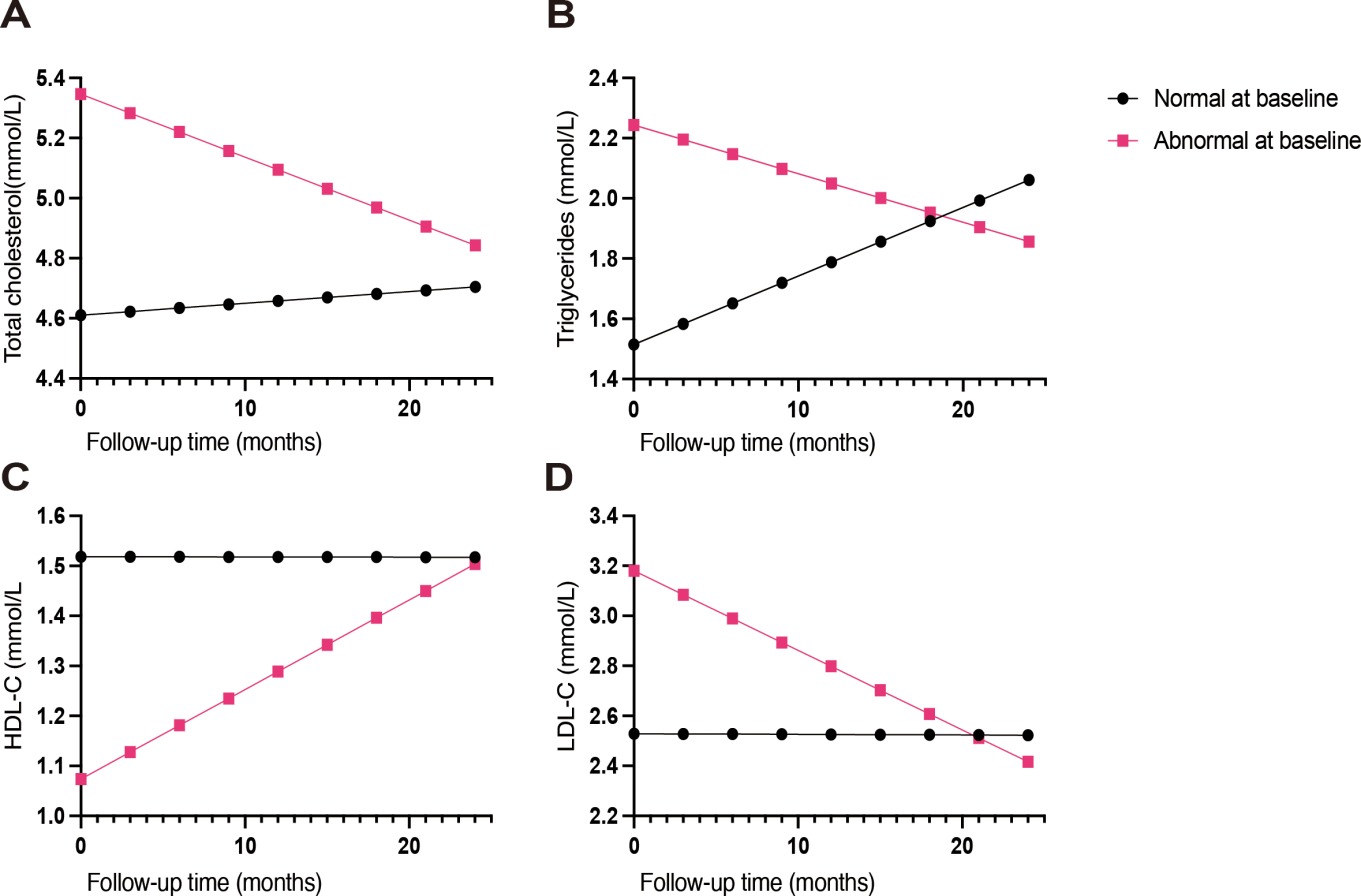
Model-predicted lipid trajectories over 24 months stratified by baseline lipid status. **(A)** Total cholesterol; **(B)** triglycerides; **(C)** HDL-C; **(D)** LDL-C. Curves show predicted mean lipid levels for normal versus abnormal baseline status (cutoffs in **Table 4**), derived from exploratory linear mixed-effects models including time (months), baseline status, and their interaction, with additional covariates as specified in the Methods. Effect estimates and 95% confidence intervals are summarized in **Supplementary Table S3**.

In exploratory linear mixed-effects models, baseline lipid status was strongly associated with subsequent lipid trajectories (**Supplementary Table S3**, **Figure 3**). For triglycerides, patients with abnormal baseline values had higher levels at baseline (β = 0.729 mmol/L; 95% CI, 0.495–0.963; *P* < 0.001) and a steeper decline over time (time × abnormal vs. normal β = −0.467 mmol/L per year; 95% CI, −0.629 to −0.305; *P* < 0.001), whereas those with normal baseline triglycerides showed an overall increase (time β = 0.273; 95% CI, 0.162–0.385; *P* < 0.001). Comparable patterns were observed for LDL-C and total cholesterol, with significant time-by-status interactions (LDL-C β = −0.379; 95% CI, −0.499 to −0.260; *P* < 0.001; total cholesterol β = −0.299; 95% CI, −0.442 to −0.155; *P* < 0.001). For HDL-C, low baseline HDL-C was associated with a lower starting level (β = −0.445 mmol/L; 95% CI, −0.723 to −0.166; *P* = 0.002) and a modest positive time interaction (β = 0.215; 95% CI, 0.035–0.396; *P* = 0.019), while the overall time effect was not significant (β = −0.001; 95% CI, −0.016–0.015; *P* = 0.94).

#### 3.4.2 Effect modification by concomitant SHD use

Concomitant use of Sanhuang Decoction (SHD) was common in this cohort (**Table 5**). Overall, 269 of 280 patients (96.1%) ever received SHD, with similar proportions across the AI+OFS, TAM, and TAM+OFS groups (94.7%, 97.2%, and 95.5%, respectively; *P* = 0.6257). At treatment initiation, 118 patients (42.1%) were taking SHD, and the prevalence of concurrent use declined over time to 84 patients (30.0%) at 24 months. At all scheduled time points from baseline to 24 months, the distribution of SHD use did not differ significantly between endocrine regimens (all *P* ≥ 0.1017).

**Table 5:** Patterns of concomitant Sanhuang Decoction (SHD) use during endocrine therapy.

Characteristic	Overall	AI+OFS	TAM	TAM+OFS	P value
Ever use of SHD, n (%)	269 (96.1%)	90 (94.7%)	137 (97.2%)	42 (95.5%)	0.6257
Never use of SHD, n (%)	11 (3.9%)	5 (5.3%)	4 (2.8%)	2 (4.5%)	0.6257
SHD use at 0 months, n (%)	118 (42.1%)	34 (35.8%)	64 (45.4%)	20 (45.5%)	0.3042
SHD use at 3 months, n (%)	129 (46.1%)	45 (47.4%)	63 (44.7%)	21 (47.7%)	0.8947
SHD use at 6 months, n (%)	114 (40.7%)	46 (48.4%)	49 (34.8%)	19 (43.2%)	0.1041
SHD use at 9 months, n (%)	114 (40.7%)	40 (42.1%)	51 (36.2%)	23 (52.3%)	0.1559
SHD use at 12 months, n (%)	114 (40.7%)	47 (49.5%)	51 (36.2%)	16 (36.4%)	0.1017
SHD use at 15 months, n (%)	106 (37.9%)	36 (37.9%)	51 (36.2%)	19 (43.2%)	0.7044
SHD use at 18 months, n (%)	98 (35.0%)	40 (42.1%)	41 (29.1%)	17 (38.6%)	0.1034
SHD use at 21 months, n (%)	96 (34.3%)	38 (40.0%)	42 (29.8%)	16 (36.4%)	0.2557
SHD use at 24 months, n (%)	84 (30.0%)	29 (30.5%)	44 (31.2%)	11 (25.0%)	0.7284

**Figure 4:**
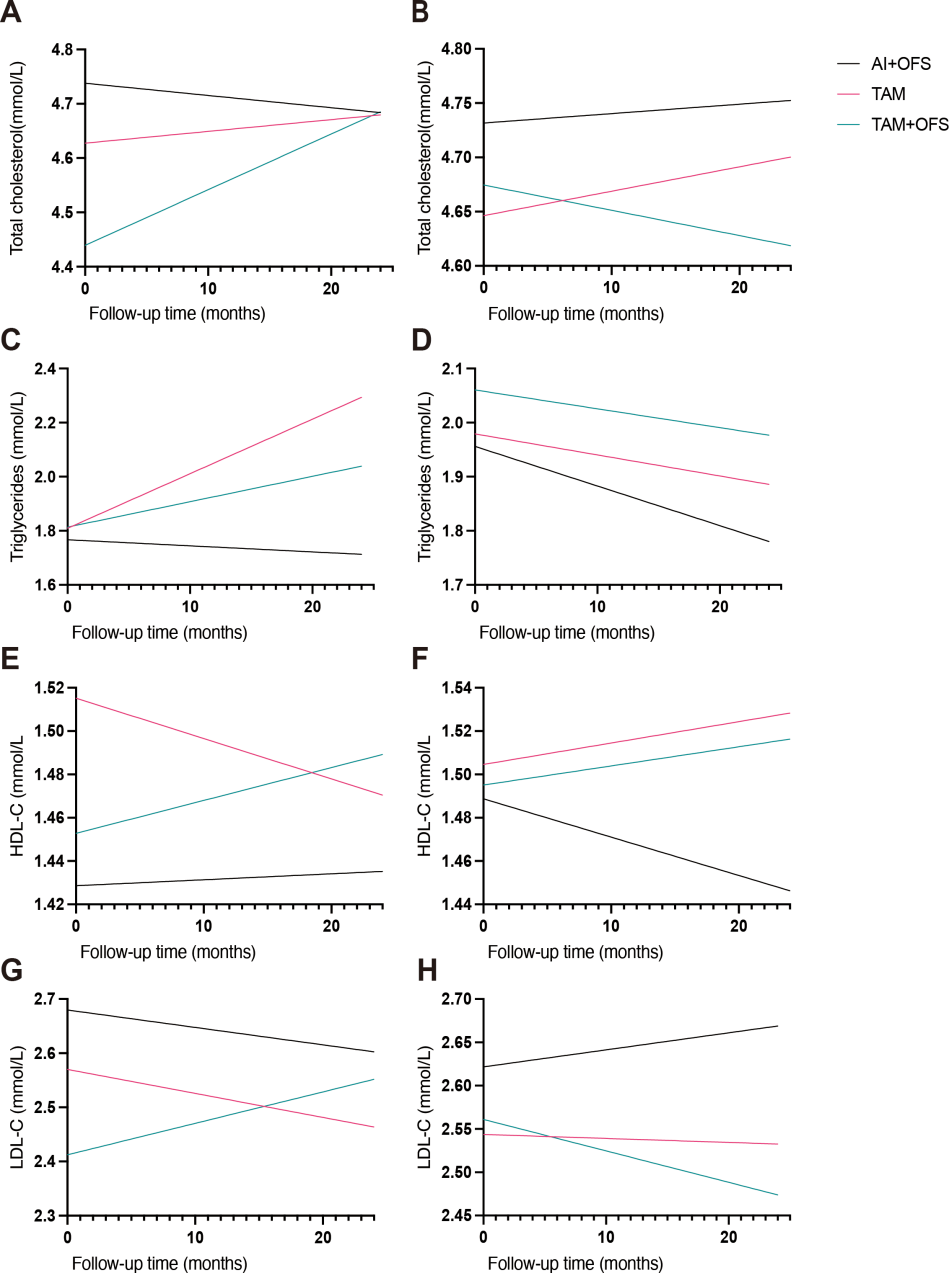
Model-predicted lipid trajectories over 24 months by concomitant SHD use and endocrine regimen. A, C, E, and G show total cholesterol, triglycerides, HDL-C, and LDL-C, respectively, among patients receiving SHD; B, D, F, and H show the corresponding trajectories among patients not receiving SHD. Each panel compares AI+OFS, TAM, and TAM+OFS. Predictions were derived from linear mixed-effects models including time (months), regimen, SHD use, and interaction terms, adjusted for baseline covariates as specified in the Methods. Interaction coefficients and 95% confidence intervals are summarized in **Supplementary Table S4**.

In exploratory linear mixed-effects models, triglyceride trajectories did not show evidence of interaction between SHD use and endocrine regimen (all interaction *P* ≥ 0.723; **Supplementary Table S4**). By contrast, several interactions were observed for cholesterol fractions. For LDL-C, the interaction between SHD use and the TAM+OFS regimen versus TAM was negative and statistically significant (β = −0.175 mmol/L; 95% CI, −0.316 to −0.034; *P* = 0.015), whereas the corresponding interaction for AI+OFS versus TAM was not significant (β = 0.032; 95% CI, −0.076 to 0.140; *P* = 0.565). A similar pattern was seen for total cholesterol, with a significant interaction for TAM+OFS versus TAM (β = −0.216 mmol/L; 95% CI, −0.405 to −0.028; *P* = 0.024) and a non-significant interaction for AI+OFS versus TAM (β = 0.025; 95% CI, −0.120 to 0.170; *P* = 0.736). For HDL-C, the interaction between SHD use and AI+OFS versus TAM was negative and statistically significant (β = −0.071 mmol/L; 95% CI, −0.132 to −0.009; *P* = 0.024), whereas the interaction for TAM+OFS versus TAM was not (β = −0.053; 95% CI, −0.133 to 0.027; *P* = 0.195). Model-predicted lipid trajectories stratified by SHD use are illustrated in **Figure 4**.

## 4 Discussion

This real-world study in predominantly premenopausal HR+/HER2− survivors suggests that AI+OFS is associated with a slightly more atherogenic lipid profile—characterized by higher model-adjusted LDL-C and lower HDL-C—and with a possible increase in the risk of new-onset carotid plaque over 24 months compared with tamoxifen-based regimens, supporting the need for careful cardiovascular surveillance and risk management (21, 22). Our work focuses on a younger, OFS-treated population often underrepresented in pivotal trials (23) and complements oncologic efficacy data from SOFT/TEXT and meta-analyses by describing longitudinal cardiometabolic safety in this demographic in greater detail (24, 25). These observations also fit within and build on our bench-to-bedside program on SHD, spanning oxidative-stress/Nrf2 signaling and clinical microenvironment modulation (19, 20, 26).

At the component level, Astragalus/astragaloside IV, Curcuma/curcumin, and Rheum/emodin each have signals for anti-inflammatory, antioxidative, and lipid-modulating activity, providing biological plausibility for the regimen-dependent SHD patterns observed in this exploratory analysis (27–29).

Mechanistically, AI+OFS produces near-total estrogen deprivation, reducing hepatic apolipoprotein A-I (ApoA-I) synthesis and thereby lowering HDL-C (30, 31); by contrast, tamoxifen’s partial hepatic estrogen agonism favors reductions in TC and LDL-C with favorable apolipoprotein changes (32, 33), consistent with the modestly higher model-adjusted LDL-C levels observed with AI+OFS compared with TAM in our mixed-effects models (β 0.088 mmol/L; 95% CI 0.013–0.163) (34). In acutely induced menopause via OFS, this differential pharmacology may be amplified, supporting the view that AI+OFS is not merely ‘absence of tamoxifen benefit’ but may be associated with an additional metabolic disadvantage in this vulnerable population (13, 35).

Beyond lipids, AI+OFS showed a higher, although imprecisely estimated, hazard of new-onset carotid plaque compared with TAM (HR 3.087; 95% CI 0.960–9.930; P = 0.059), with lower plaque-free survival in Kaplan–Meier analysis (log-rank P = 0.041), a pattern consistent with reports of greater carotid plaque and cardiovascular events with AIs versus tamoxifen (36, 37). Although the wide confidence interval suggests sparse-event instability, the direction is biologically coherent: profound estrogen loss drives endothelial dysfunction, oxidative stress, and impaired HDL-mediated reverse cholesterol transport, which may promote foam-cell formation and plaque progression (38–40). In young women, new plaque is often considered to be non-calcified and lipid-rich (‘vulnerable’), which may heighten long-term risk (41).

Baseline metabolic status contributed little as an independent predictor of new-onset or worsening fatty liver, whereas higher baseline HbA1c was independently associated with earlier initiation of lipid-lowering therapy (HR 2.537; 95% CI 1.290–4.987; P = 0.007) and showed a borderline association with new-onset carotid plaque (HR 2.579; 95% CI 0.987–6.741; P = 0.053), supporting guideline-directed baseline risk stratification before cancer therapy initiation (35). Conceptually, ET may act as a ‘second hit’ on pre-existing metabolic susceptibility, and our exploratory analyses of baseline lipid status showed that patients with abnormal baseline lipids had higher starting levels but steeper subsequent declines, whereas those with normal baseline lipids tended to show small increases over time (**Supplementary Table S3**), warranting vigilance even in patients with apparently “normal” baseline lipids.

Regimen-dependent effects of SHD were suggested in the interaction models: the SHD use × (TAM+OFS vs. TAM) term was associated with lower model-adjusted LDL-C (β −0.175 mmol/L; 95% CI −0.316 to −0.034; P = 0.015) and total cholesterol (β −0.216 mmol/L; 95% CI −0.405 to −0.028; P = 0.024) with TAM+OFS relative to TAM among SHD users (**Supplementary Table S4**). While translation to clinical event risk is beyond the scope of this study, this pattern is directionally consistent with data relating LDL-C lowering to improved outcomes and with our prior randomized work suggesting that SHD can modulate inflammatory and microenvironmental markers during endocrine therapy (20, 22). Conversely, the SHD use × (AI+OFS vs. TAM) interaction for HDL-C was negative (β −0.071 mmol/L; 95% CI −0.132 to −0.009; P = 0.024), raising the possibility of a context-dependent effect of SHD under near-complete estrogen suppression, whereas tamoxifen’s hepatic partial agonism may permit a more favorable interaction; however, these exploratory findings require cautious interpretation and external validation (20, 26, 36).

While a marked regression-to-the-mean effect related to baseline lipid status was observed, treatment-related differences in model-adjusted HDL-C (AI+OFS vs. TAM) and the signal toward a higher hazard of new-onset plaque with AI+OFS persisted after multivariable adjustment. These patterns are compatible with treatment-related effects beyond statistical regression but should be interpreted cautiously given the limited number of plaque events and the exploratory nature of several interaction analyses, and they highlight potential cardiovascular risk even when baseline lipids appear “normal.”

Strengths include a real-world design, focus on a younger OFS-treated cohort, and longitudinal modeling of lipid trajectories with adjustment for key confounders, alongside exploratory assessment of regimen-dependent SHD effects. Limitations include retrospective design, modest sample size for subgroups, small numbers of arterial plaque and SHD-related events, 24-month follow-up that precludes hard-event assessment, EMR-based endpoint ascertainment with potential detection bias, potential residual confounding by indication owing to the non-random allocation of endocrine regimens, and non-standardized SHD composition; these warrant cautious interpretation and replication.

Clinically, our findings support routine lipid monitoring and cardiovascular risk assessment for patients starting AI+OFS, irrespective of baseline lipids, consistent with contemporary cardio-oncology guidance (35). Shared decision-making between AI+OFS and TAM+OFS should weigh oncologic benefit (25) against individualized cardiometabolic risk, and early referral to cardio-oncology/preventive cardiology may be considered in higher-risk patients (42). Prospectively, multicenter cohorts with standardized serial vascular imaging are needed to quantify plaque dynamics under AI+OFS (13, 43), and mechanistic as well as interventional studies should test whether early lipid-lowering or other cardioprotective strategies mitigate risk in this setting.

## 5 Conclusion

In this real-world cohort of premenopausal HR-positive/HER2-negative breast cancer survivors, AI+OFS was associated with modestly higher LDL-C, lower HDL-C, and a trend toward increased risk of new-onset carotid plaque compared with tamoxifen-based regimens. Concomitant SHD use showed regimen-dependent associations with lipid profiles—appearing more favorable with TAM+OFS than with AI+OFS—and these exploratory findings support individualized cardiovascular monitoring and cautious, tailored use of adjunctive SHD.

## Data Availability

The original contributions presented in the study are included in the article/**Supplementary Material**. Further inquiries can be directed to the corresponding author.
