# Supplementary material for: Endocrine regimen and early subclinical atherosclerosis in premenopausal HR+/HER2− breast cancer: real-world evidence and regimen-dependent effects of Sanhuang Decoction
Source: Front Oncol. 2026 Jan 28;16:1695776. doi: 10.3389/fonc.2026.1695776 (PMC12890639; doi:10.3389/fonc.2026.1695776)
Supplement: Supplementary file 1 [file Table1.docx]

**Table S1.** Multivariable linear mixed-effects model estimates for longitudinal serum lipid profiles according to endocrine therapy regimen

| **Lipid outcome** | **Effect** | **β (95% CI)** | **P value** |
| --- | --- | --- | --- |
| Triglycerides (mmol/L) | Group: AI+OFS vs. TAM | -0.026 (-0.220 to 0.168) | 0.794 |
|  | Group: TAM+OFS vs. TAM | 0.049 (-0.196 to 0.294) | 0.694 |
|  | Time (per year) | 0.051 (-0.031 to 0.133) | 0.219 |
|  | Time × (AI+OFS vs. TAM) | -0.111 (-0.240 to 0.018) | 0.092 |
|  | Time × (TAM+OFS vs. TAM) | -0.026 (-0.193 to 0.142) | 0.766 |
| LDL-C (mmol/L) | Group: AI+OFS vs. TAM | 0.088 (0.013 to 0.163) | 0.021 |
|  | Group: TAM+OFS vs. TAM | -0.060 (-0.154 to 0.034) | 0.211 |
|  | Time (per year) | -0.022 (-0.048 to 0.005) | 0.111 |
|  | Time × (AI+OFS vs. TAM) | 0.022 (-0.020 to 0.063) | 0.311 |
|  | Time × (TAM+OFS vs. TAM) | 0.025 (-0.030 to 0.079) | 0.373 |
| HDL-C (mmol/L) | Group: AI+OFS vs. TAM | -0.046 (-0.086 to -0.007) | 0.022 |
|  | Group: TAM+OFS vs. TAM | -0.034 (-0.085 to 0.016) | 0.179 |
|  | Time (per year) | 0.001 (-0.014 to 0.016) | 0.903 |
|  | Time × (AI+OFS vs. TAM) | -0.011 (-0.035 to 0.013) | 0.352 |
|  | Time × (TAM+OFS vs. TAM) | 0.016 (-0.016 to 0.047) | 0.325 |
| Total cholesterol (mmol/L) | Group: AI+OFS vs. TAM | 0.091 (-0.006 to 0.189) | 0.067 |
|  | Group: TAM+OFS vs. TAM | -0.071 (-0.194 to 0.051) | 0.254 |
|  | Time (per year) | 0.028 (-0.008 to 0.064) | 0.123 |
|  | Time × (AI+OFS vs. TAM) | -0.031 (-0.087 to 0.026) | 0.284 |
|  | Time × (TAM+OFS vs. TAM) | 0.009 (-0.064 to 0.083) | 0.8 |

**Table S2.** Linear mixed-effects models for longitudinal lipid profiles according to endocrine therapy regimen

|  | **New-onset fatty liver** | | **Initiation of lipid-lowering drugs** | | **New-onset arterial plaque** | |
| --- | --- | --- | --- | --- | --- | --- |
|  | **HR (95% CI)** | **P-value** | **HR (95% CI)** | **P-value** | **HR (95% CI)** | **P-value** |
| **covariate** |  |  |  |  |  |  |
| **Group: AI+OFS vs. TAM** | 0.652 [0.351–1.213] | 0.177 | 1.920 [0.882–4.180] | 0.100 | 3.087 [0.960–9.930] | 0.059 |
| **Group: TAM+OFS vs. TAM** | 1.490 [0.770–2.884] | 0.237 | 1.662 [0.619–4.462] | 0.314 | 2.235 [0.533–9.368] | 0.271 |
| **Baseline age (per year)** | 0.999 [0.962–1.037] | 0.957 | 1.022 [0.975–1.072] | 0.360 | 1.003 [0.930–1.081] | 0.943 |
| **Baseline BMI (per kg/m²)** | 1.049 [0.959–1.148] | 0.293 | 0.992 [0.889–1.106] | 0.883 | 0.978 [0.832–1.148] | 0.783 |
| **Baseline HbA1c (per %)** | 0.610 [0.277–1.341] | 0.219 | 2.537 [1.290–4.987] | 0.007 | 2.579 [0.987–6.741] | 0.053 |

**Table S3.** Effect of baseline lipid status on longitudinal lipid trajectories in linear mixed-effects models

| **Lipid outcome** | **Analysis** | **Effect** | **β (95% CI)** | **P value** |
| --- | --- | --- | --- | --- |
| Triglycerides (mmol/L) | Baseline TG status | Time (per year) | 0.273 (0.162 to 0.385) | <0.001 |
|  | Baseline TG status | Baseline status × abnormal vs. normal | 0.729 (0.495 to 0.963) | <0.001 |
|  | Baseline TG status | Time (per year) × Baseline status × abnormal vs. normal | -0.467 (-0.629 to -0.305) | <0.001 |
| LDL-C (mmol/L) | Baseline LDL-C status | Time (per year) | -0.003 (-0.029 to 0.024) | 0.838 |
|  | Baseline LDL-C status | Baseline status × abnormal vs. normal | 0.652 (0.455 to 0.848) | <0.001 |
|  | Baseline LDL-C status | Time (per year) × Baseline status × abnormal vs. normal | -0.379 (-0.499 to -0.260) | <0.001 |
| Total cholesterol (mmol/L) | Baseline TC status | Time (per year) | 0.047 (0.011 to 0.083) | 0.011 |
|  | Baseline TC status | Baseline status × abnormal vs. normal | 0.735 (0.505 to 0.966) | <0.001 |
|  | Baseline TC status | Time (per year) × Baseline status × abnormal vs. normal | -0.299 (-0.442 to -0.155) | <0.001 |
| HDL-C (mmol/L) | Baseline HDL-C status | Time (per year) | -0.001 (-0.016 to 0.015) | 0.94 |
|  | Baseline HDL-C status | Baseline status × abnormal vs. normal | -0.445 (-0.723 to -0.166) | 0.002 |
|  | Baseline HDL-C status | Time (per year) × Baseline status × abnormal vs. normal | 0.215 (0.035 to 0.396) | 0.019 |

**Table S4.** Interaction between concomitant SHD use and endocrine regimen in linear mixed-effects models for lipid outcomes

| **Outcome** | **Interaction term** | **Effect (95% CI)** | **P value (formatted)** |
| --- | --- | --- | --- |
| Total Cholesterol | SHD use × (AI+OFS vs. TAM) | 0.025 [-0.120, 0.170] | 0.736 |
|  | SHD use × (TAM+OFS vs. TAM) | -0.216 [-0.405, -0.028] | 0.024 |
| Triglycerides | SHD use × (AI+OFS vs. TAM) | -0.019 [-0.346, 0.308] | 0.907 |
|  | SHD use × (TAM+OFS vs. TAM) | -0.077 [-0.502, 0.348] | 0.723 |
| LDL-C | SHD use × (AI+OFS vs. TAM) | 0.032 [-0.076, 0.140] | 0.565 |
|  | SHD use × (TAM+OFS vs. TAM) | -0.175 [-0.316, -0.034] | 0.015 |
| HDL-C | SHD use × (AI+OFS vs. TAM) | -0.071 [-0.132, -0.009] | 0.024 |
|  | SHD use × (TAM+OFS vs. TAM) | -0.053 [-0.133, 0.027] | 0.195 |
